# Immunoglobulin G4 (IgG4)-Related Sialadenitis and Dacryoadenitis With Chronic Rhinosinusitis

**DOI:** 10.7759/cureus.9756

**Published:** 2020-08-15

**Authors:** Samar Aboulenain, Tatiana P Miquel, Juan J Maya

**Affiliations:** 1 Internal Medicine, University of Miami Miller School of Medicine Palm Beach Regional Campus, Atlantis, USA; 2 Internal Medicine, JFK Medical Center, Atlantis, USA; 3 Pathology, JFK Medical Center, Atlantis, USA; 4 Rheumatology, JFK Medical Center, Atlantis, USA

**Keywords:** igg4 related sialadenitis, immunoglobulin type g4, sjogren's, rhinosinusitis, kuttner’s tumor, mikulicz’s disease

## Abstract

Immunoglobulin G4-related disease (IgG4-RD) is a new disease entity of rare and complex immune-mediated fibroinflammatory conditions that can affect any organ. The concomitance of IgG4 sclerosing sialadenitis and dacryoadenitis with rhinosinusitis is extremely rare. We report a case of IgG4 sclerosing sialadenitis and dacryoadenitis (Mikulicz’s disease) diagnosed in a middle-aged African American man with a long-standing history of chronic rhinosinusitis who presented with progressively worsening bilateral salivary and lacrimal glands swelling. Imaging revealed pansinusitis, symmetric enlargement of the lacrimal glands, parotid glands, and submandibular glands. Serological IgG4 level was significantly elevated and the diagnosis of IgG4 sclerosing sialadenitis was confirmed by histopathology. A robust clinical response in the facial swelling and nasal manifestations was noted after the initiation of immunotherapy with corticosteroids.

## Introduction

Immunoglobulin G4-related disease (IgG4-RD) is a recently recognized immune-mediated fibroinflammatory systemic condition that often mimics other disease processes, such as malignancy or granulomatous diseases. The most affected organ is the pancreas and it is the first organ to be recognized as an IgG4-related disease. In 2003, Kamisawa et al. detected a predominance of IgG4-positive plasma cells in association with CD4 and CD8 T lymphocytes in the tissue biopsy of patients with autoimmune idiopathic pancreatitis identifying IgG-associated pancreatitis as a new entity of diseases [1].

In addition to the pancreas (Type 1 autoimmune pancreatitis), IgG4-related disease can potentially affect nearly any organ including biliary system, salivary glands, lacrimal glands, eyes (orbital pseudotumor), lymph nodes, retroperitoneum, large vessels, thyroid gland, lungs, pleura or kidneys. It can be multi-centric in its distribution or isolated to a single organ.

In this article, we report a rare presentation of IgG4 sclerosing sialadenitis and dacryoadenitis causing a rapidly progressive swelling of the head and neck in a patient with a chronic history of chronic rhinosinusitis.

## Case presentation

A 46-year-old African American man was transferred to our facility for an ear-nose-throat (ENT) evaluation of a progressively worsening swelling of the head and neck. His symptoms started with painless enlargement of the right submandibular glands nine months ago, followed by swelling of the left submandibular gland and bilateral parotid glands six months later. Most recently, he noticed bilateral eyelid swelling associated with excessive lacrimation and diplopia for the past two months. The patient reported a 17-year history of rhinorrhea, nasal congestion, frontal headaches and hyposmia that is refractory to conservative management. A review of systems was remarkable for unintentional weight loss and intermittent pruritic erythematous maculopapular rash that mostly appears on his both arms and spontaneously disappears. He denied fever, arthralgias, dry mouth, dry eyes, nose crusting, epistaxis, change in the nose shape, wheezing, cough or shortness of breath.

In the past year, he had two recent hospitalizations for acute bronchitis treated with bronchodilators and antibiotics. He denied smoking, alcohol consumption or illicit drug use. He denied any past surgical history and current medications. He has worked in the construction industry for the past 30 years and denied any family history of rheumatological conditions.

**Figure 1:**
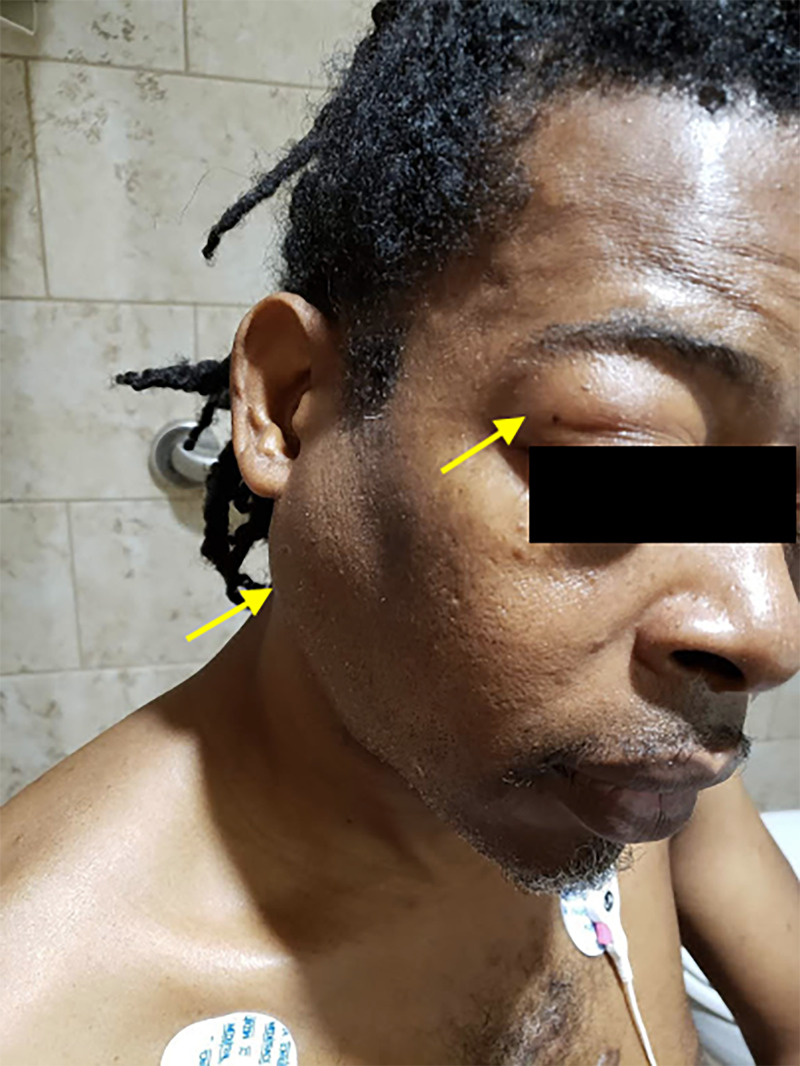
Lacrimal gland (upper yellow arrow) and parotid gland (lower yellow arrow) enlargement.

On physical examination, he appeared comfortable with normal body temperature, normal heart rate, respiratory rate and blood pressure. The parotid, submandibular and lacrimal glands were firm, non-tender and symmetrically enlarged on both sides (Figure 1). Bilateral swelling of the eyelids and ptosis were noted. The visual acuity and the function of the extraocular muscles were intact. When compared to previous photographs of himself, no proptosis or exophthalmos was appreciated. No rashes, arthritis or lymphadenopathy were noted.

Initial laboratory workup was remarkable for an elevated erythrocyte sedimentation rate (ESR) (34 mm/hr) and eosinophilia (8%). C-reactive protein (CRP) was found to be within normal limits (0.4 mg/dL). Serological antibodies testing was negative for antinuclear antibody (ANA), rheumatoid factor, double-stranded DNA, anticentromere, anti-Ro (SSA) and anti-La (SSB), anti-Scl-70 and ribonucleoprotein (RNP). Additional serological testing included negative serum fungal antibodies and normal serum angiotensin-converting enzyme level. Serum immunoglobulin G class 3 and 4 were elevated at >227 mg/dL (normal value 15-102 mg/dL) and 1541 mg/dL (normal value 2-96 mg/dL), respectively.

**Figure 2:**
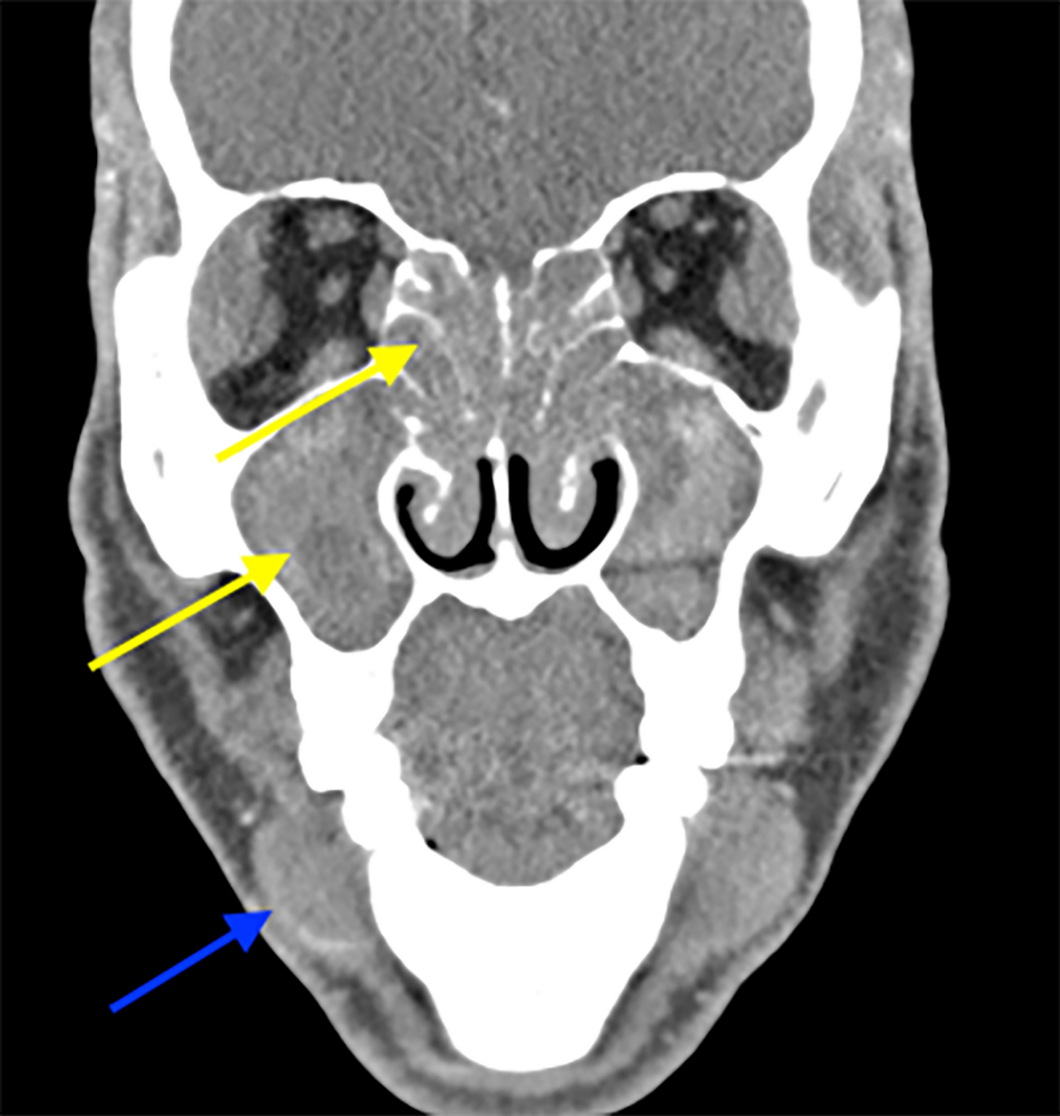
Coronal plane of computed tomography (CT) scan of the face demonstrating bilateral obliteration of ethmoid (upper yellow arrow) and maxillary sinuses (lower yellow arrow). Bilateral submandibular gland enlargement (blue arrow).

Computed tomography (CT) scan of the head with contrast revealed opacification of the bilateral maxillary, sphenoid, ethmoid and frontal sinuses, and enlargement of the bilateral lacrimal, parotid and submandibular glands (Figure 2).

**Figure 3:**
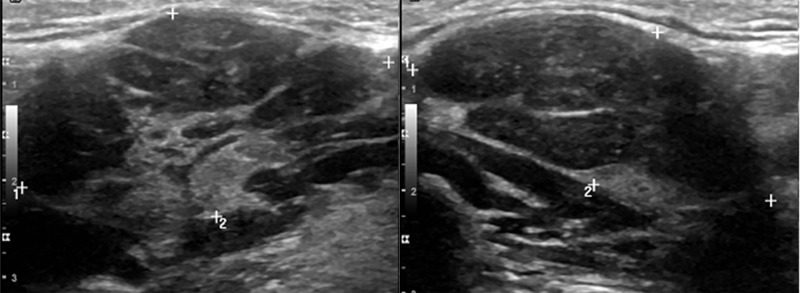
Ultrasound of the neck reveals enlargement of the right submandibular gland measuring 4.0 x 2.2 cm and the left submandibular gland measuring 2.7 x 1.6 cm.

Ultrasound (US) of the neck demonstrated bilateral submandibular gland soft tissue edema without gross evidence of drainable fluid collection (Figure 3).

**Figure 4:**
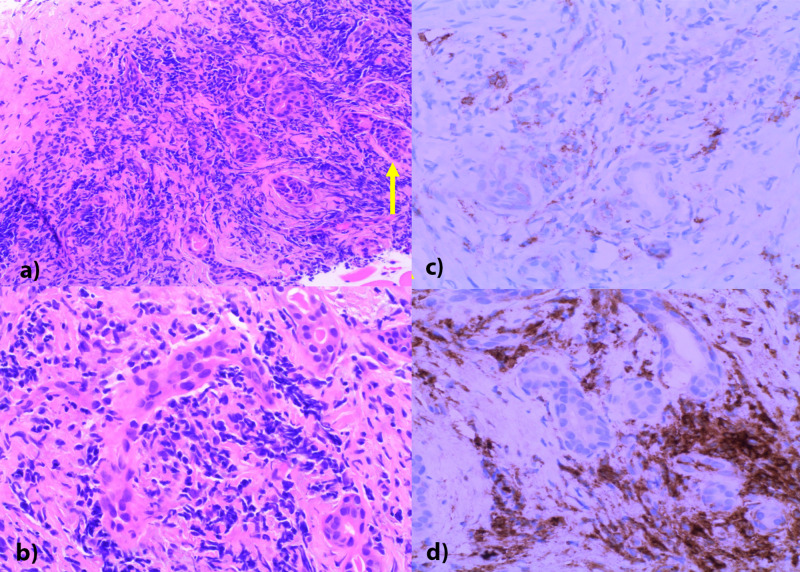
(a) Low power (10X) H&E stain showing fibrotic parotid tissue with prominent lymphoplasmacytic infiltrate. Ductal structures are obliterated by inflammation and fibrosis (yellow arrow). (b) High power demonstrating an infiltration of crushed lymphocytes in a background of dense fibrosis. (c) CD138 immunostaining highlighting scattered plasma cells. (d) IgG-4 stain highlighting IgG4-bearing plasma cells representing the majority of all CD138-positive plasma cells and representing approximately 70-80% of plasma cells.

The patient underwent a US-guided core biopsy of the left submandibular gland. Biopsy demonstrated atrophic salivary gland tissue with a chronic inflammatory infiltrate and extensive fibrosis. A CD138 stain highlighted several plasma cells and IgG4 highlighted a majority of these cells with an increase in the background staining (Figure 4).

The diagnosis of IgG4 sclerosing sialadenitis was reached based on the constellation of symptoms, the histopathological findings and the elevated serum IgG4 concentration. Chronic rhinosinusitis and dacryoadenitis were believed to be due to IgG4-related disease. The diplopia was presumed to be secondary to the lacrimal gland edema inducing a displacement to the orbit. Treatment was initiated with corticosteroids with a significant improvement in facial swelling, rhinitis, hyposmia and diplopia. He was discharged home to continue prednisone at a dose of 20 mg every 12 hours to follow up in the rheumatology clinic.

## Discussion

IgG4-related disease is a cluster of rare and complex immune-mediated fibroinflammatory conditions that can affect nearly any organ. Despite the disease is named after IgG4, there is growing evidence that IgG4 is in fact not the inflammatory mediator rather than a response to a primary inflammatory process provoked by type 2 helper and regulatory T-cells via interleukin-4, interleukin-10, interleukin-12 and interleukin-13. Mattoo et al. detected an expansion of IL-1β, TGF-β1 and IFN-γ secreting CD4+ cytotoxic T-lymphocytes in peripheral blood and IgG4-associated inflammatory lesions, all believed to be secreted by T-helper and T-regulatory cells [2]. Although the pathogenesis of the disease is not yet fully elucidated, potential mechanisms are autoimmunity, genetic predisposition and triggered by bacterial infection through molecular mimicry [3].

IgG4-related disease most commonly affects males with a male to female ratio around 3:1 and the mean age ranges around 60 years [4]. IgG4 sclerosing sialadenitis and dacryoadenitis is a rare condition that is also known as Mikulicz’s disease. In the past, it is believed that it was misdiagnosed as seronegative Sjogren’s disease or granulomatous diseases such as sarcoidosis or granulomatosis with polyangiitis. In our case, the diagnosis of Sjogren’s disease was unlikely due to the absence of secretory dysfunction and negative SSA and SSB. Sarcoidosis was also unlikely due to the lack of classical histopathological features.

Ophthalmic involvement in IgG4-RD including dacryoadenitis is not an uncommon association. The most common structure affected is the lacrimal gland and is estimated to be around (62%-68%) of all patients with ophthalmic IgG4-RD. Dacryoadenitis is commonly associated with salivary gland swelling, similar to what we experienced in our case [5,6].

Nasal obstruction and anosmia/hyposmia are the most commonly present nasal manifestations in IgG4-RD. It is more common in males than females with a ratio of 3:1. Imaging classically reveals bilateral thickening of the affected sinuses and nasal obstruction. Complications are not uncommon, and it may involve the orbit causing proptosis and eyelid swelling. Patients with IgG4-RD and concomitant chronic rhinosinusitis are more likely to have elevated serum IgG4 and eosinophilia which were present in our case [7-9].

The diagnosis of IgG4-RD is challenging, and a high index of suspicion is necessary. The abundant infiltration of lymphocytes with plasma cells expressing IgG4 in immunohistostaining and storiform fibrosis is pathognomonic. However, neither histopathological findings nor a clinical picture alone is sufficient to make the diagnosis. The most accurate diagnostic tool is based on the clinical, serological and radiological findings altogether. Serum IgG4 level may be elevated with a sensitivity of 87.2% and a specificity of 82.6%. Imaging modalities using CT, CT with positron emission tomography (PET), or magnetic resonance (MR) imaging can be used to guide the management. When IgG4-RD is suspected a core or open biopsy is warranted to rule out malignancy and IgG4-RD mimickers. Fine needle aspiration alone is often inconclusive. The exclusion of other conditions that can mimic IgG4-RD clinical picture or histopathology is essential prior to reaching the diagnosis of IgG4-RD [10,11].

An international consensus guidance statement on the management and treatment of IgG4-RD recommends corticosteroids as first-line therapy. The initiation of a steroid-sparing agent is recommended to avoid the long-term side effects of corticosteroids and to prevent the risk of relapse. It is recommended for patients who are in remission to continue maintenance therapy which may consist of low-dose glucocorticoids or a steroid-sparing agent including rituximab, cyclophosphamide, azathioprine or mycophenolate. In asymptomatic patients who have limited disease, watchful waiting can be adopted [10].

The prognosis of IgG4-RD is yet to be fully understood. However, it is considered to be relatively favorable with a 10-year overall survival rate of 89.0% [4]. The majority of the patients respond well to corticosteroids; however, the risk of recurrence is common after discontinuation of therapy. A few observational studies suggest an increased risk of malignancy in these patients; however, the results are conflicting [12-14]. More studies are needed to further understand the nature and prognosis of this disease.

## Conclusions

The IgG-related disease is a new entity of immune-mediated fibroinflammatory conditions that can be a rare etiology of any mass-like lesions. The diagnosis should be based on clinical findings, serological and pathological findings. The clinical manifestation usually responds drastically to treatment with corticosteroids. Other therapeutic options including rituximab can be used in refractory or recurrent disease.
